# Filter feeding in devil rays is highly sensitive to morphology

**DOI:** 10.1098/rspb.2024.2037

**Published:** 2025-01-22

**Authors:** Shirel R. Kahane-Rapport, Julia Teeple, James C. Liao, E. W. M. Paig-Tran, James A. Strother

**Affiliations:** ^1^ Old Dominion University, 5115 Hampton Boulevard, Norfolk, VA 23529, USA; ^2^ California State University Fullerton, 800 State College Boulevard, Fullerton, CA 92867, USA; ^3^ Whitney Laboratory for Marine Bioscience, Department of Biology, University of Florida, 9505 North Ocean Shore Boulevard, St Augustine, FL 3208, USA

**Keywords:** manta ray, fish filtration, suspension feeding, bio-inspired design, CFD, PIV

## Abstract

Mobulid rays (manta and devil rays) use a highly specialized filtering apparatus to separate plankton food particles from seawater. Recent studies have indicated that captive vortices form within the microscale pores of the filter, which enhance filtration efficiency through a novel mechanism referred to as ricochet separation. The high throughput and clog resistance of this filtration process have led to the development of several bioinspired engineered filtration systems. However, it is still unclear how changes to the filter morphology influence the surrounding flow patterns and filtration efficiency. We address this question by examining the flow fields around and filtering properties of mobulid filters with systematically varied morphologies, using a combination of computational fluid dynamics and experiments on physical models. While the pore size is the principal determinant of filtration efficiency in a sieve filter, we found that the captive vortices in a mobulid filter grow or shrink to fill the pore, and changes in the pore size have modest effects. By contrast, the filtration efficiency appears to be highly sensitive to the orientation of the filter lobes (microscale plate-like structures). These results provide a foundation for interpreting the morphological differences between species and also for generating optimized bioinspired designs.

## 1. Introduction

Capturing and ingesting food from the water column, or suspension feeding, is a foraging mechanism used by a wide diversity of animals, from 1 μm protists to 30 m blue whales [1–4]. On the smallest scale, suspension feeding enables low mobility or sessile aquatic organisms to capture food from the surrounding fluid medium [5]. By contrast, larger, highly mobile suspension feeders can exploit abundant, albeit often patchy, planktonic prey [6–9]. The largest vertebrate suspension feeding animals are sometimes referred to as ‘mega’ filter feeders and include mobulid rays (e.g. manta rays and other devil rays), whale sharks, paddlefishes, oarfishes, megamouth sharks, basking sharks and baleen whales. Although these animals display a range of feeding-specific behaviours (engulfment feeding, suction feeding and ram feeding), the general process consists of drawing water into the buccal cavity, filtering out solid food particles such as phytoplankton or zooplankton, expelling the excess water and ingesting the food particles into the oesophagus.

**Figure 1.**
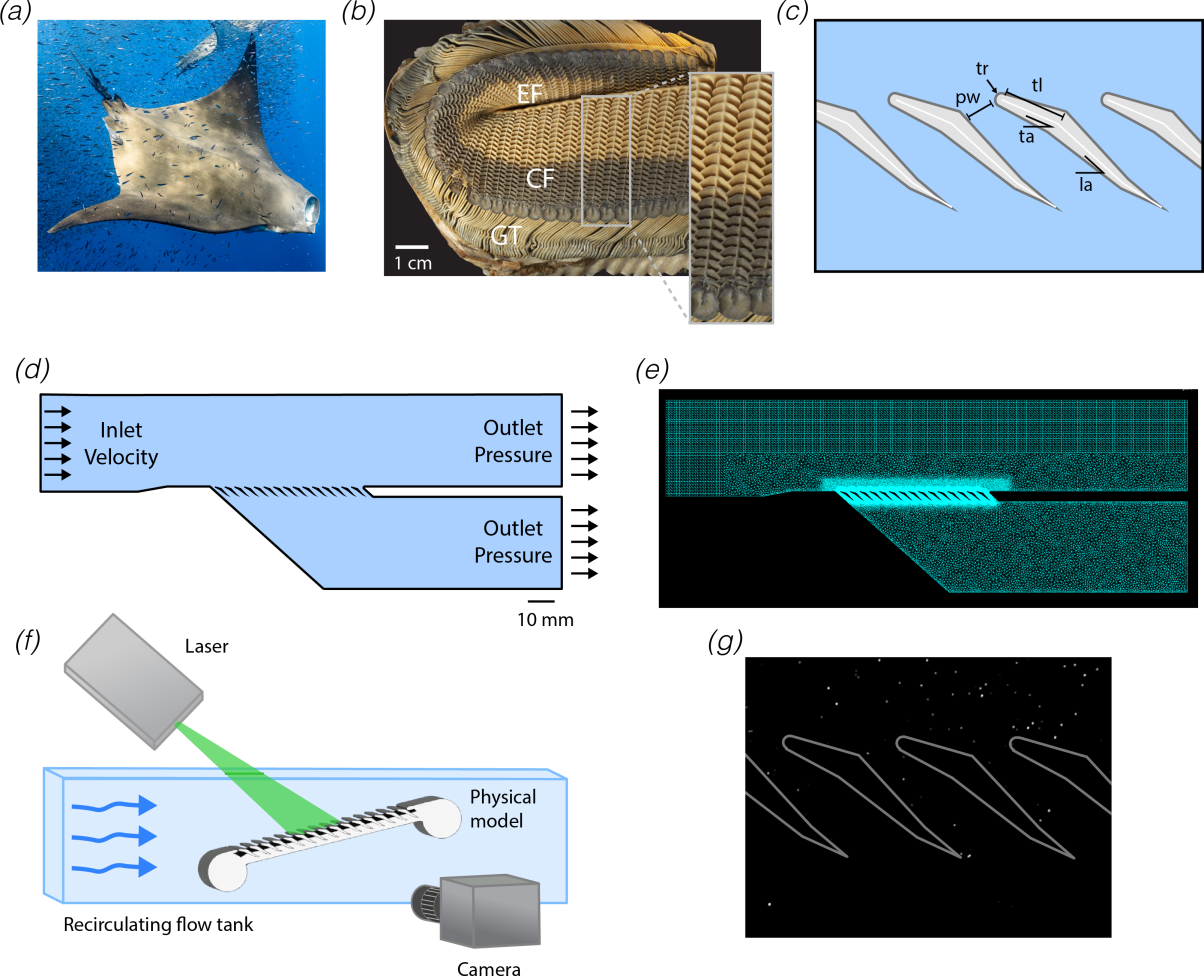
Computational and experimental approach used to examine flow around mobulid filters. (*a*) Photo of feeding *Mobula tarapacana* (credit: Henley Spiers). (*b*) Photo of excised gill arch from *M. tarapacana* showing anterior-facing filtering structure (inset: magnified view). (*c*) Diagram of cross-section through filter lobe array in wing-like orientation, indicating the morphological parameters examined in this study. (*d*) Schematic diagram of computational domain for simulations of flow around mobulid filters. These simulations aim to understand the microscale flow around an individual filter lobe but include an array of 15× lobes and large inlet/outlet channels to reduce edge effects. Flow velocities and filtration efficiencies were computed for the tenth lobe in the array, unless otherwise indicated. (*e*) Triangular mesh used for computational fluid dynamics model. (*f*) Schematic diagram of experimental apparatus used to record the flow fields around physical models of mobulid filters using digital particle image velocimetry (DPIV). (*g*) Representative DPIV image after pre-processing. GT, gill tissue; EF, epibranchial filter; CF, ceratobranchial filter; pw, pore width; tr, tip radius; tl, tip length; ta, tip angle; la, lobe angle.

Most mega filter feeders employ highly specialized feeding structures to collect solid food particles from large volumes of water. Whale shark filters are composed of flattened, reticulated, fused filter meshes that sit flush to the dorsal and ventral surface of the buccopharynx [10,11]. In baleen whales, the filtering structure is comprised primarily of stiffened keratin plates that fray into fringes at the terminal ends [12]. Paddlefish and basking shark rakers have similarly elongate morphologies, though basking shark rakers exhibit additional fine scale surface wrinkling and a tapered trailing edge [13–15]. Mobulid rays have a filtering apparatus with a morphology distinct from that observed in other mega filter feeders (figure 1*a,b*). The filter surface is keratinous and attaches to the anterior and posterior surfaces of the branchial arches [11]. The filters are comprised of arrays of paired, plate-like filter lobes that are angled in relation to the incoming flow. The morphology of the filter is complex and has been documented in several species using anatomical and histological studies [7,11,16].

Early work on suspension feeding suggested that the majority of animals employed some form of hydrosol filtration or sieve filtration. In a hydrosol filter, solid particles are captured on adhesive surfaces within the filter structure. By contrast, sieve filters capture particles on pores smaller than the particle diameter [4]. A conventional cross-flow filter uses a variant of sieve filtration, in which the incoming fluid runs tangential to the sieve filter in order to reduce the bulk accumulation of material on the filter surface or cake formation [17]. However, recent work has suggested that many suspension feeding animals employ more complex filtration mechanisms. Physical modelling studies examining paddlefish and basking sharks have shown that vortices develop between the gill arches and reduce cake formation on neighbouring sieve filters, which has been referred to as vortical cross-step filtration [15,18,19].

Despite their large size and ecological importance [20], much remains unknown regarding the behaviours and biomechanics of filter feeding in mobulid rays. Manta and devil rays typically perform ram filter feeding, in which the animals swim through plankton blooms with an open mouth, continuously separating food particles from the incoming water. Gut content analysis indicates that mobulid rays consume a diversity of prey items, but their diet is largely euphausids and copepods [20–22]. Mechanistic studies examining mobulid rays have indicated that they employ an unusual filtration process. The unique morphology of the filter lobes induces the formation of captive vortices that form in the filter pore. These vortices produce flow fields in which fluid streamlines are compacted against the filter surfaces [7]. Solid particles following these streamlines contact the filter surfaces and are pushed toward streamlines that no longer pass though the filter pore, in a process that has been referred to as ricochet filtration. This mechanism enables high-throughput filtration of particles substantially smaller than the filter pore size. The described process has parallels to direct interception filtration, a form of hydrosol filtration in which particles following fluid streamlines are captured when the streamline passes within one particle radius of an adhesive surface [4]. This mechanism also has similarities to lateral displacement filtration, a low-Reynolds number (Re < approx. 50) filtration process employed in microfluidics engineering in which a fluid is passed through a grid of posts [23]. Solid particles follow streamlines through this grid and make contact with the posts, which pushes them to one side of the grid. Recent computational studies examining filtration in American shad have also suggested a filtration mechanism with similarities to both lateral displacement filtration and ricochet separation [24].

The mobulid filtering apparatus has inspired the development of numerous high-efficiency engineered filtration systems. Since the initial discovery of ricochet separation in 2018, there have been several studies that have applied manta-inspired filtration to both small and large-scale applications. At small scales, the mobulid filtration structure inspired the development of microfluidics filters that separate cell-like particles with near 100% efficiency [25,26]. Similarly, bio-inspired filters created via aligned electrospun nanofibrous silk fibroin membrane have been used to successfully filter oil from water [27]. On a larger scale, bio-inspired devices using ricochet mechanisms have been proposed for combating marine microplastic pollution [28] and as a mechanism to successfully separate sand particles from water [29]. Bio-inspired air filters based on mobulid filters have successfully filtered particulate matter down to 1 µm from the air [30].

Despite these advances, there are still substantial gaps in our understanding of the mechanics of filter feeding in mobulid rays. In particular, the morphology of the filtering apparatus varies dramatically between mobulid species, but very little is known about the functional implications of these changes in morphology. In this study, we systematically examine how changes in the morphology of the filtering apparatus impact the flow fields around the filter and the filtration efficiency. Computational fluid dynamics (CFD) is used to model the flow fields around filters with modified pore widths, angles relative to the incoming flow and leading-edge shape. The results from these computational studies are then validated using physical models in combination with digital particle image velocimetry (DPIV). Lastly, we estimate how the observed changes in flow patterns alter particle filtration efficiency using particle tracing simulations.

## 2. Methods

### (a) Numerical simulations

A series of simulations were performed to understand how filter lobe morphology influences the flow around and performance of a small section of the filter. Morphological parameters measured from *Mobula tarpacana* were used to construct a two-dimensional parametric geometric model of a filter lobe (figure 1*c*; [7]). A small block of the filtering apparatus was generated by creating an array of filter lobes (15 lobes) in a virtual test chamber, which included a channel carrying a tangential freestream flow above the filter lobes and a filtrate channel carrying filtered water below the lobes (figure 1*d*). The flow field around the filter lobes was simulated using a laminar CFD solver [7]. Although the precise structure of the flows within the buccal cavity is not yet clear, the arrangement of the gill arches within the buccal cavity suggests that the filter lobes on the posterior surfaces of the gill arch encounter flow in one direction (wing-like orientation), while the lobes on the posterior surfaces encounter flow in an opposing direction (spoiler-like orientation). All simulations were performed with filter lobes in both wing-like and spoiler-like orientations (figure 1*c*). The boundary conditions of the model were configured to mimic the local flow conditions around the filter lobes in a freely swimming animal (tangential flow velocity: 0.3 m s^−1^; transverse velocity: 10 mm s^−1^; [7]). The transverse velocity was controlled by varying the pressure at the outlet of the filtrate channel (−1 to −30 Pa for spoiler-like; −1 to −10 Pa for wing-like; 10 values) and selecting the pressure that produced the nearest transverse velocity (figure 1*d*). These pressures used for wing-like filter lobes spanned a biologically realistic range as estimated in a prior study [7], but the filter lobes in the spoiler-like orientation showed robust characteristics across this range and so a larger window was examined.

The model geometry was discretized using an all-triangular mesh, and the flow was then simulated using a laminar flow solver based on the finite-element method (Adina 9.8.1). The mesh was generated using Delaunay triangulation (figure 1*e*). The element sizes were fixed on the boundary (20 μm at solid surfaces, 1 mm on channel periphery) and refined using an explicitly coded size function (1.5 mm thick boundary layer around each filter lobe surface, 20 μm at surface, increasing to 150 μm), yielding a mesh with approximately 400 000 elements. Preliminary simulations with coarser meshes produced nearly identical flow patterns and mean flow rates through the filter apparatus, and subsequent simulations used the finer mesh density to ensure robust convergence. The element formulation used flow-condition interpolation and a solution was found using a sparse solver. A time-dependent simulation was employed to enable the freestream velocity to be slowly ramped up to its prescribed value to aid convergence (formulation: composite integration; ramp: 200 s; steps: repeating sequence of 1 × 10 s followed by 10 × 1 ms). However, in all cases the flow appeared to approach a steady-state solution.

After the flow field was computed, the trajectory of solid spherical particles carried by the water was simulated using rigid body equations of motion based on forces derived from the fluid velocity, acceleration and vorticity at the particle position. This model includes the pressure gradient, acceleration reaction, drag, Saffman’s lift and contact forces [7]. The equations of motion were solved using an explicit Runge–Kutta–Fehlberg method implemented using custom-written C programs that used functions from the GNU Scientific library (2.6−150200.3.4.3) and libMesh library (1.6.2). To avoid edge effects, particle tracing simulations examined solid-fluid separation for particles at a single pore in the centre of the array (pore after the tenth lobe). The transverse flow velocity was found by computing the mean flux through the exit pore. Next, the streamlines entering into this pore were computed by uniformly seeding the exit area of the pore with virtual particles (50×) that were neutrally buoyant and infinitesimal, then tracing these to the upstream boundary using time reversed equations of motion. Virtual finite-sized solid particles (50×) were then released from the found upstream area and the trajectory that each particle followed was computed. The solid particles were classified as excluded if they remained above the filter for the distance of at least the starting pore and the two subsequent pores. The filtration efficiency was calculated as the number of excluded particles divided by the total number of particles.

This approach was used to examine the flow patterns around and filtration efficiency for a broad array of filter morphologies and flow conditions centred around the observed values. We varied the pore width, lobe angle, lobe tip angle, lobe tip length and lobe tip radius (see the electronic supplementary material, table S1). When varying the lobe angles and lobe tip parameters, the pore width was maintained fixed by adjusting the array period. For each morphology, particle tracing simulations were used to calculate the filtration efficiency for neutrally buoyant particles in a range of sizes (0.02–1.1 mm, 18 values). In total, 1000 CFD simulations and 18 000 particle tracing simulations were performed.

We then examined how changes in the flow environment around the filtering apparatus altered the filtration process. To simulate the filter feeding process at different swimming speeds, we examined the measured filter lobe morphology at varied tangential freestream flow velocities (0.01–0.5 m s^−1^, 10 values). The methodology employed for CFD and particle tracing simulations was similar to that used for examining different morphologies. However, to ease interpretation, for these simulations the pressure at the filtrate outlet was held constant rather than varied to maintain a constant transverse flow velocity. Scripts and model files have been deposited in a public repository (Dryad, doi: 10.5061/dryad.280gb5n0s [31]).

### (b) Digital particle image velocimetry experiments

A physical model of the *M. tarapacana* filter structure was constructed using previously measured morphometrics [7], mirroring the geometry used for the CFD simulations described above. The model was designed using Autodesk Inventor 2023 and printed at 5× scale using a Formlabs printer in white resin. Variants of this model were then constructed using similar methods (pore width increased from 1.1 to 2.1 mm, lobe angle was increased or decreased by 10°).

DPIV was used to determine the flow fields that develop around the filtering structure (figure 1*f*–*g*). Each model was positioned in the working section of a small custom-built recirculating acrylic flume (working-section: 5 cm × 6 cm × 23 cm). The water was seeded with silver-coated hollow glass microspheres (Potters Industries, 13 μm diameter, 1.60 g cc^−3^), and illuminated using a laser (OptoEngine LLC, 532 nm, 2 W) equipped with lightsheet generating optics (10° Powell lens). The microspheres were then recorded using a high-speed camera (Edgertronic SC1, Canon EX-Sigma 105 mm lens, 768 × 768 pixels, 1000 frames s^−1^). The lightsheet was positioned 4 mm from the lateral edge of the model to reduce edge effects and obscuration from out-of-plane material. To illuminate the narrow pore between the filter lobes, the laser was aligned to run parallel to the filter lobes, and this was re-adjusted for each set-up. Using this approach, it was possible to visualize the flow field at the opening of the filter pore for every configuration, although shadowing occasionally obscured deeper portions of the filter pore. The filter lobes were painted matte black to reduce glare.

The flow patterns around the filter lobes are expected to be strongly influenced by the tangential and transverse velocity of the freestream flow above the filtering structure. However, these velocities are difficult to precisely control within an experimental context. To resolve this issue, we examined the flow fields at two freestream flow velocities (50 and 100 mm s^−1^) and for filters positioned with three different orientations relative to the freestream flow (3°, 6° and 12°). A post hoc analysis based on DPIV results was then used to select experiments with tangential velocities and tangential-to-transverse velocity ratios approximating the estimated *in vivo* values (as Reynolds scaled values, measured geometry: 160 mm s^−1^ and 1 : 12 (wing), 190 mm s^−1^ and 1 : 15 (spoiler); enlarged pore width: 150 mm s^−1^ and 1 : 21 (wing), 190 mm s^−1^ 1 : 26 (spoiler); −10° angle: 180 mm s^−1^ and 1 : 13 (wing), 200 mm s^−1^ and 1 : 23 (spoiler); +10°: 160 mm s^−1^ and 1 : 24 (wing), 220 mm s^−1^ and 1 : 14 (spoiler)). Overall, the DPIV simulations and CFD measurements are both intended to replicate the flow conditions in the buccal cavity of freely swimming animals, but they use somewhat different approaches driven by the constraints of each methodology. Since both approaches ultimately yield the same freestream and transverse velocities, the flow patterns at the filter pore are expected to be similar.

DPIV analysis was conducted using the Open Source Image Velocimetry package (2.2.0). Masks were generated using a custom MATLAB script. Images were pre-processed using histogram equalization and difference of Gaussian filtering. Cross-correlation was performed using direct correlation computation with ensemble averaging (18 × 18 windows, 66% overlap, 500 frame pair ensemble average, 20 000 frames per sequence). Preliminary analyses indicated that standard DPIV algorithms lack the dynamic range to accurately capture both the fast-moving tangential flow and slow-moving recirculating flow. To resolve this issue, we employed a high dynamic range DPIV strategy in which the cross-correlation was calculated using a multitude of frame pair intervals (14 values, 1−28 frame offset) and a composite flow field was then constructed using the interval yielding the strongest signal at a given location (post-processing of displacements implemented in custom MATLAB script, similar to [32]). All calculated velocities were Reynolds-scaled based on the 5× scale of the physical models.

## 3. Results and discussion

### (a) Filtration is insensitive to increases in pore width

**Figure 2.**
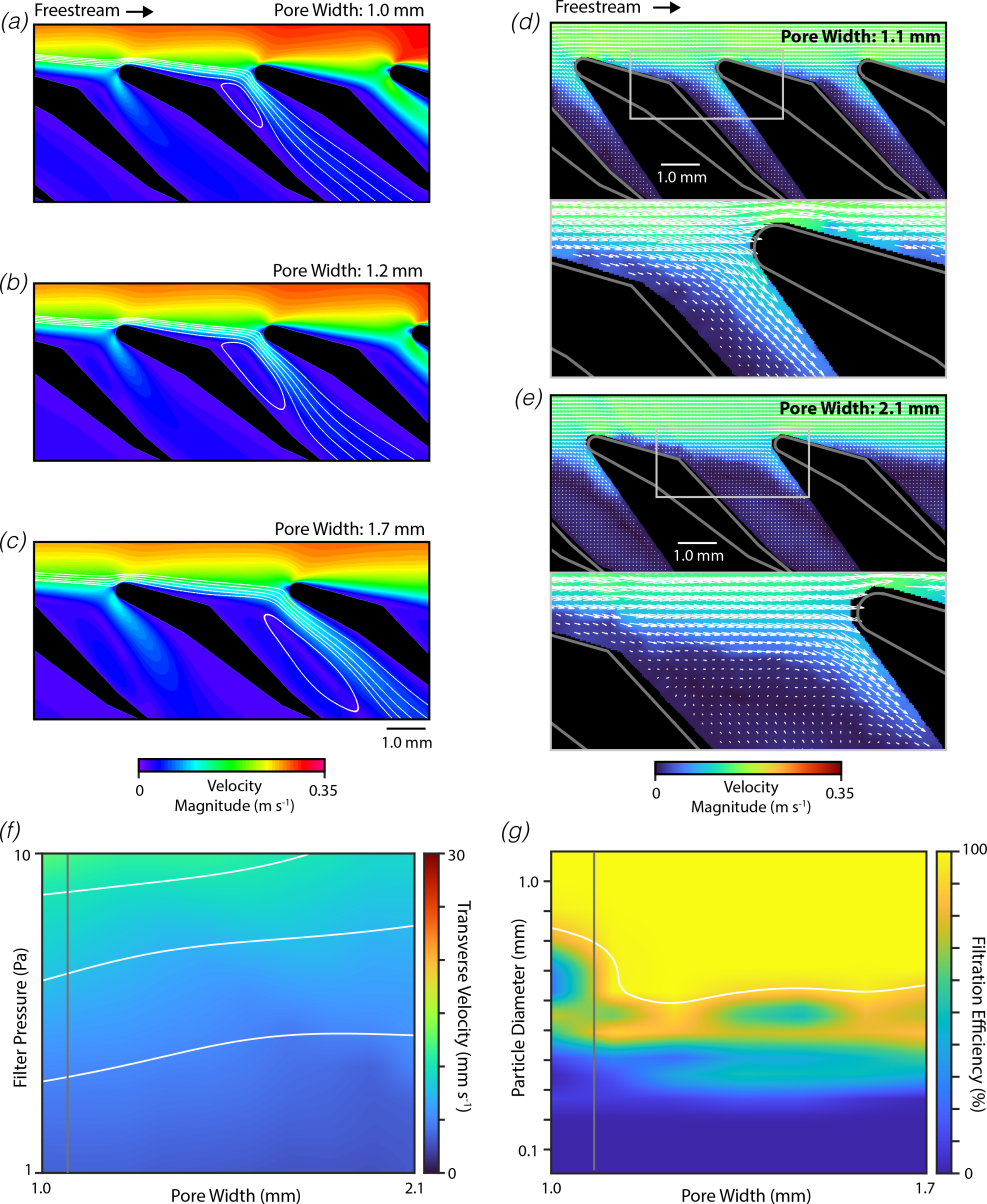
Filters in wing-like orientation are robust to variation in pore width. (*a–c*) Representative depictions of flow through mobulid filter (flow from left to right, background: velocity magnitude, streamlines: white lines) from a series of CFD simulations examining morphologies with varying pore widths (actual for *M. tarapacana*: 1.1 mm). (*d–e*) Flow fields around physical models of mobulid filters with varied pored widths measured using DPIV (bottom: magnified view of region in top panel indicated by rectangle). (*f*) Flow rate through the filter measured by the transverse velocity as a function of the pressure across the filter and the pore width (actual: vertical grey line, contours: white lines). (*g*) Filtration efficiency as a function of the tested solid particle diameter and the pore width (actual: vertical grey line, 90% cut-off: white line, transverse velocity: 10 mm s^−1^).

In sieving solid–fluid separation, the filtration efficiency decreases sharply for particle sizes smaller than the pore width. The dimensions of the pore also vary substantially between mobulid species [16]. To examine how pore width alters filtration efficiency in the mobulid filtering apparatus, we constructed a series of CFD models in which the pore width was varied around the measured morphology (measured: 1.1 mm, examined: 1.0–2.1 mm; figure 2*a*–c). For these models, we found that water flowing over the lobes in a wing-like orientation resulted in flow separation at the convex bend on the downstream surface of the filter lobe near the pore, and the formation of a captive vortex within the pore. The magnitude of the flow velocity within the captive vortex was comparatively small, such that fluid in this vortex was near-stagnant. Water entered the filter pore through a narrow region at the downstream side of the filter pore, just upstream of the leading edge of the adjacent filter lobe. This result is consistent with findings from a prior study [7]. Surprisingly, we found that the qualitative size of the primary vortex scaled with the pore width, shrinking as the pore width decreased and growing as the width increased. Consequently, the region through which fluid passes into the filter tended to remain constant even as the pore width changed.

To examine these findings empirically, we used DPIV to record the flow fields around three-dimensional-printed physical models positioned in a flow tank (figure 2*d*–*e*). Similar to our CFD simulations, the measured flow fields indicated that flow separation occurred at the convex bend, which results in the formation of an adjacent area with recirculating flow and near-stagnant velocities. It was not possible to experimentally resolve the full recirculation zone owing to the proximity of solid surfaces, but the remainder of the flow field and inspection of the raw image data suggest a flow pattern similar to that observed in CFD simulations. The measured flow fields also indicate that fluid passes over this recirculating zone before entering the filter pore through a region of fast-moving fluid on the downstream side of the pore. Furthermore, we found that physical models with an increased pore width had an enlarged stagnant region, such that the region through which water enters into the filter pore remained relatively constant even as the pore width changes. However, some differences were observed between the CFD simulations and DPIV measurements, such as reduced boundary thickness above the filter lobes in the DPIV results. This most likely results from minor differences in the imposed flow conditions between these two systems (see Methods).

Given this overall agreement between the CFD and DPIV results, CFD modelling was then used to systematically explore the effects of varying pore widths. To understand how changes in the morphology affect the volumetric flow rate through the filter, we calculated the mean transverse velocity as a function of the pressure drop over the filter and pore width (figure 2*f*). As might be expected, increases in the filter pressure resulted in increases in the transverse velocity (represented by increasingly warm colours along the *y*-axis of figure 2*f*). However, increases in the pore width yielded only modest effects on the transverse velocity (represented by similar colours along the *x*-axis and horizontal contour lines in figure 2*f*). These properties may be summarized by computing the hydrodynamic permeability of the filter, which is defined as the transverse velocity normalized by the pressure drop and then multiplied by the fluid viscosity [33]. The hydrodynamic permeability only decreased by 19% when the pore width was set to double the measured value (1.0 vs. 2.1 mm, 91% increase).

We then computed the filtration efficiency as a function of the particle diameter and pore width (figure 2*g*). The cut-off particle diameter was taken as the diameter yielding 90% filtration efficiency, and the cut-off diameter for the measured morphology was calculated as 795 μm. Contrary to what would be expected for sieving filtration, this cut-off point was only weakly dependent on the pore width, even decreasing to 650 μm when the pore width was approximately doubled (1.0 vs 1.7 mm).

**Figure 3.**
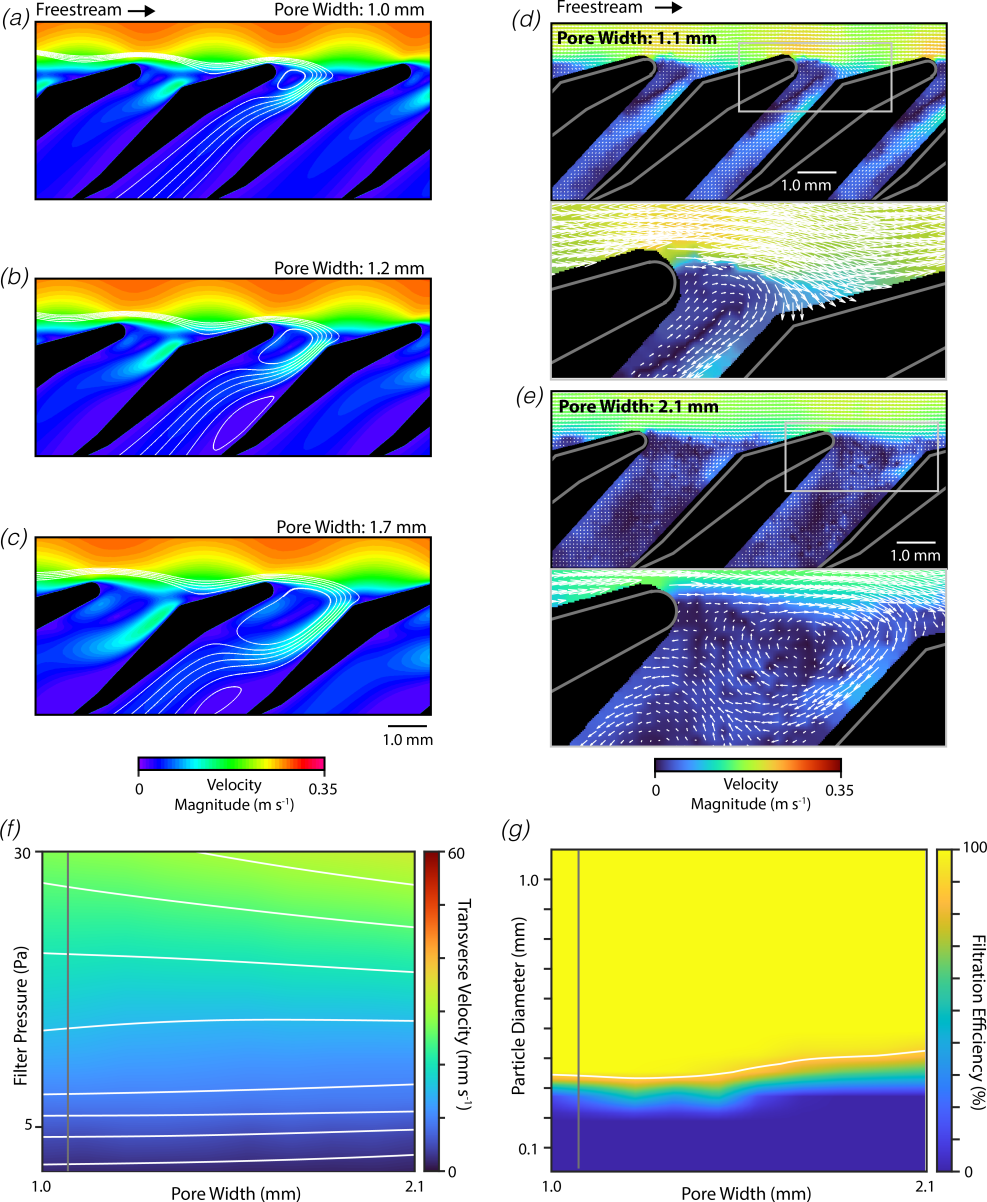
Filters in spoiler-like orientation are also insensitive to pore width variation. Similar to figure 2 except shown for filters in the spoiler-like orientation.

Similar simulations were performed with the filter lobe oriented in the spoiler-like orientation relative to the tangential flow (figure 3*a*–*c*). Despite the substantial differences between the wing-like and spoiler-like orientations, we observed several parallels in the resulting flow patterns. For the measured morphology, we found that flow separation occurred just downstream of the tip of the filter lobe, producing a ‘primary’ captive vortex within the pore (figure 3*b*). The CFD simulations also predict the formation of a ‘secondary’ captive vortex deeper within the pore. Flow velocities within these captive vortices were low, and water passed into the filter pore through a narrow region of fast-moving flow on the downstream side of the pore. Increases in the pore width were found to increase the qualitative size of both vortices, such that the region through which water entered the pore remained relatively constant. To provide further validation of these results, we used DPIV to record the flow fields around physical models of the filter lobes under similar flow conditions (figure 3*d*–*e*). The measured flow fields mirror the results of the CFD simulations, indicating the formation of a primary vortex within the filter pore, with water entering the filter through a narrow region of fast-moving fluid on the downstream side of the pore.

A series of CFD models were then used to examine the effect of pore width on flow through the filter and filtration efficiency. Here too, the pore width had only modest effects on the transverse velocity through the filter (figure 3*f*). The permeability decreased by 9% when the pore width was increased to double the measured morphological value (1.0. vs 2.1 mm, at 10 mm s^−1^ transverse velocity). We also observed that the cut-off diameter was relatively independent of the pore width, increasing by 20% when the pore width was approximately doubled (1.0 vs 1.7 mm; figure 3*g*). Relative to the wing-like orientation, we found that spoiler-like orientation had lower permeability (40% lower at measured morphology) and higher filtration efficiency (cut-off diameter of 330 μm at measured morphology).

These simulations indicate that wing-like and spoiler-like orientation offer contrasting advantages and disadvantages, with the wing-like orientation providing greater permeability and the spoiler-like orientation enabling greater filtration efficiency for smaller particles. This difference in performance was not noted in previous studies [7], likely as a result of where in the array measurements were made, as will be subsequently examined.

These results also demonstrate that the filtration efficiency of the mobulid filtering structure is insensitive to the pore width, in strong contrast to what would be expected for a conventional sieve filter. These findings suggest that the prey selectivity of different mobulid species is unlikely to be well predicted by morphological measurements of the pore width of the filtering structure, and that such predictions require a more nuanced understanding the biomechanics of filter feeding in each species. In addition, these data indicate that pore width may not be a key parameter for optimization in bioinspired engineered filter systems.

### (b) Filter lobe angle modulates captive vortex position

**Figure 4.**
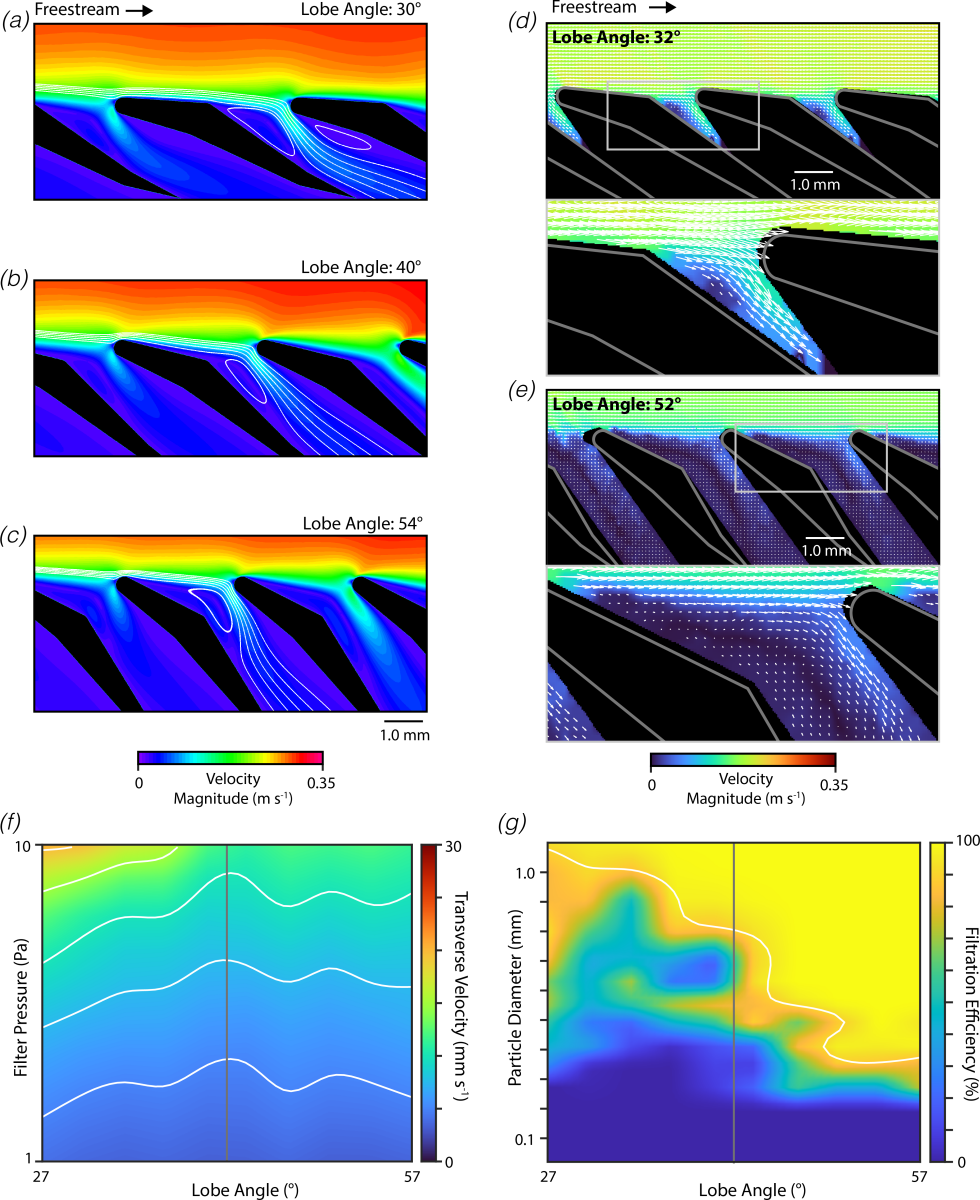
Filters in wing-like orientation are sensitive to lobe angle. (*a–c*) Representative depictions of flow through mobulid filter (flow from left to right, background: velocity magnitude, streamlines: white lines) from a series of CFD simulations examining morphologies with varying lobe angles (actual for *M. tarapacana*: 42°). (*d–e*) Flow fields around physical models of mobulid filters with varied lobe angles measured using DPIV (bottom: magnified view of region in top panel indicated by rectangle). (*f*) Flow rate through the filter measured by the transverse velocity as a function of the pressure across the filter and the lobe angle (actual: vertical grey line, contours: white lines). (*g*) Filtration efficiency as a function of the tested solid particle diameter and the pore width (actual: vertical grey line, 90% cut-off: white line, transverse velocity: 10 mm s^−1^).

We next examined how changes in the filter lobe angle alter the mechanics of filter feeding. We constructed a series of CFD models of filters in wing-like orientation with lobe angles around the measured value (measured: 42°, examined: 27°– 57°; figure 4). The computed streamlines indicated that decreases in the lobe angle tended to cause the captive vortex to shift deeper into the pore, while increases in the lobe angle tended to cause this captive vortex to move to a more superficial position (figure 4*a*–*c*). Furthermore, for filter lobes near the measured morphology, there is substantial streamline redirection and compaction at the leading-edge surface of the filter lobe (figure 4*b*). This redirection and compaction are central to the ricochet separation mechanism, as it enables surface contact that pushes the solid particles further into the freestream flow. However, this redirection and compaction appeared to be reduced in morphologies with smaller lobe angles. The distance from the furthest pore-bound streamline to the filter apex was 56% greater with a lobe angle of 30° compared with a lobe angle of 54°. The effects of lobe angle on the flow field around the filter lobes were also studied using DPIV experiments conducted with physical models, and our findings largely parallel those from the CFD simulations (figure 4*d*–*e*). The measured flow fields indicate that increases in the lobe angle tend to cause the captive vortex to shift to a more superficial position within the pore.

To further understand these effects, we examined the transverse velocity as a function of the filter pressure and lobe angle using CFD simulations (figure 4*f*–*g*). The transverse velocity for wing-like filters was lowest for lobe angles near to the measured value, decreasing as the lobe angle was either increased or decreased away from the measured value (figure 4*f*). Relative to the measured morphology, the permeability increased by 60% when the lobe angle was reduced by 15° and the permeability increased by 4% when the angle was elevated by 15°. We also examined how changes in the lobe angle alter the filtration efficiency of filter lobes operating in wing-like orientation (figure 4*g*). We found that the filtration efficiency was strongly affected by the lobe angle, with the cut-off particle diameter decreasing 2.8× when the lobe angle was changed from 27° to 57°. Unexpectedly, we observed complex non-monotonic variations in the filtration efficiency with particle diameter and lobe angle. This may reflect complex interactions between streamline compaction, changes in flow pattern along the length of the array and the ricochet separation mechanism.

**Figure 5.**
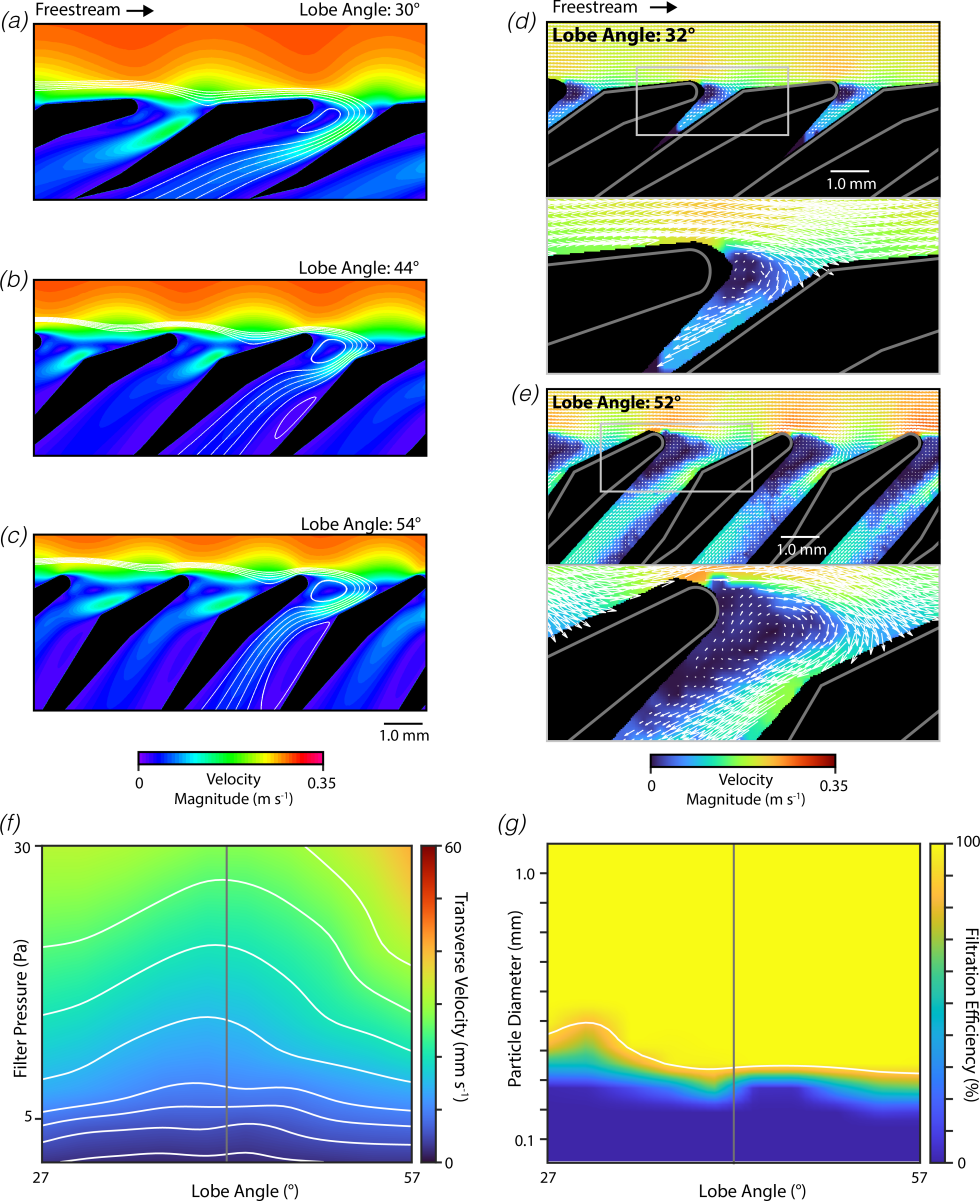
Filters in spoiler-like orientation are robust to lobe angle. Similar to figure 4 except shown for spoiler-like orientation.

We proceeded to examine how changes in the lobe angle affect the flow and filtration dynamics for filter lobes in the spoiler-like orientation (figure 5). Despite the differences in the overall flow patterns around wing-like and spoiler-like filters, shifts in the lobe angle had similar effects. For filter lobes in the spoiler-like orientation, decreases in the lobe angle drove the primary captive vortex deeper into the filter pore, while increases in the angle caused the captive vortex to move to a more superficial location (figure 5*a*–c). For lobe angles near to the measured value, the centre of the vortex was situated at the point of maximum constriction between adjacent filter lobes. Furthermore, decreases in the lobe angle reduced the streamline redirection and compaction that occurred on the upstream surfaces of the filter lobe. The distance from the furthest computed pore-bound streamline to the filter apex was 51% greater in geometries with a lobe angle of 30° compared with those with a lobe angle of 54°. DPIV experiments were again used to record the flow fields around similar physical models, and the measured flow fields reciprocate the findings from CFD simulations (figure 5*d*–e).

To further understand these effects, we examined the transverse velocity as a function of the filter pressure and lobe angle (figure 5*f*). Our results indicate that transverse velocity is lowest at lobe angles near the measured value, increasing if the lobe angle is increased or decreased. Relatedly, the hydrodynamic permeability is lowest near the measured lobe angle of 42°, increasing by 37% if the angle is reduced by 15° and by 47% if the angle is elevated by 15°. We then explored the functional consequences of these changes in the flow patterns by computing the filtration efficiency as a function of the particle diameter and lobe angle (figure 5*g*). We found that the filtration efficiency was moderately dependent on the lobe angle, gradually improving as the lobe angle increased. The cut-off particle diameter was 37% greater for the geometry with a 27° lobe angle compared with the geometry with a 57° lobe angle. This improvement in filtration efficiency may be associated with the observed increase in streamline compaction at the upstream surfaces. Overall, these results suggest that the lobe angle is a key morphological parameter in determining the flow patterns around and the performance of the mobulid filter.

Understanding the effects of morphological parameters on functional performance may provide some insights into the selective pressures that have shaped these structures. The filtration efficiency of wing-like filters appears less robust than that of spoiler-like filters, as changes of a few degrees in lobe angle produce dramatic changes in the efficiency of wing-like filters. If robustness were the only factor of consequence, then filter morphologies that avoid wing-like configurations might be expected. The greater hydrodynamic permeability of the wing-like filter lobes may offset these effects, though other unknown aspects of the morphology, biomechanics, or development may be influencing this outcome. Similarly, these simulations indicate that the measured morphology is non-optimal for both filtration rate and filtration efficiency. Relative to the measured morphology, our results indicate that morphologies with higher lobe angles would result in greater filtration rates at the same pressure (or less drag at the same filtration rate) and also increased particle capture efficiency. These results demonstrate the need for further studies examining the anatomy and biomechanics of these complex filtering structures.

### (c) Effects of lobe angle are largely mediated by lobe tip

Given the pronounced effects of the lobe angle on flow through the filter array and the filtration efficiency of the apparatus, we next explored whether these effects were caused by the entire filter lobe or whether they resulted principally from the interaction of the lobe tip with the freestream flow. To address this question, we constructed a series of simulations in which the body of the filter lobe was held constant at the measured angle while the tip of the filter lobe was varied (measured: 22°, examined: 7–37°).

We first examined the effects of varying the lobe tip angle in filters with the wing-like orientation (electronic supplementary material, figure S1). Our results indicated that varying the angle of just the tip of the filter lobe reproduced many of the same effects that were observed when varying the angle of the entire filter lobe (electronic supplementary material, figure S1 compared with figure 4). In particular, our results indicate that the hydrodynamic permeability was also lowest near the measured morphology, increasing for lower and higher lobe tip angles (71% increase at 7°, 33% increase at 37°, compared with measured 22°). We found that the filtration efficiency was moderately sensitive to the lobe tip angle, with the efficiency increasing as the lobe tip angle was shifted away from the measured value (28% decrease in cut-off diameter at 7° and 57% decrease in diameter at 37°, compared with measured 22°).

We then examined the filter lobes in the spoiler-like orientation (electronic supplementary material, figure S2). As above, we found that varying the lobe tip angle reproduced many of the effects observed when varying the angle of the entire filter lobe, with some exceptions (electronic supplementary material, figure S2 compared with figure 5). The hydrodynamic permeability was lowest near the measured morphology, and increased for lower and higher lobe tip angles (24% increase at 7° and 39% increase at 37°, compared with measured 22°). Similarly, the filtration efficiency improved as the lobe tip angle was increased, with the cut-off particle diameter decreasing by 30% as the tip angle was changed from 7° to 37°.

Overall, these results suggest that the angle of the tip of the filter lobe relative to the freestream flow has a central role in determining the efficiency of the filtration process.

### (d) Lobe tip shape influences flow and filtration

To further understand how the shape of the filter lobe influences the flow patterns that develop around the filter lobe, we next implemented a series of simulations varying the length of the filter lobe tip (measured: 2.0, examined: 0.75–4.0 mm). For filter lobes in the wing-like orientation, we found that changes in the tip length had few effects on the qualitative features of the flow around the lobes (electronic supplementary material, figure S3A–C). Despite yielding qualitatively similar flow patterns, increases in the tip length produced large decreases in the hydrodynamic permeability of the structure (65% decrease for 4.0 mm lobe tip compared with 1.0 mm; electronic supplementary material, figure S3D). Furthermore, changes in the lobe tip length had major effects on the filtration efficiency (2.1× increase in cut-off diameter for 2.4 mm tip length, compared with 1.0 mm length; electronic supplementary material, figure S3E).

For filter lobes in the spoiler-like orientation, we found that changes in the lobe tip length also did not have substantial effects on the qualitative features of the flow but did alter specific critical aspects of the flow pattern. Increases in the tip length tended to cause the stagnation point (point where right-most pore-bound streamline contacts lobe) to shift position downstream (electronic supplementary material, figure S4A–C). Furthermore, varying the tip length tended to produce changes in the shape of the constriction between the primary captive vortex and the adjacent filter lobe (visible as bright patches of fast-moving flow in this area in the electronic supplementary material, figure S4A–C), with increases in the tip length tending to produce elongation of this constriction. These changes in flow may be responsible for observed changes in the hydrodynamic permeability of the structure (electronic supplementary material, figure S4D), which decreased 43% as the tip length was extended from 1.0 to 4.0 mm. Nonetheless, this change in the hydrodynamic permeability was not mirrored by changes in the estimated filtration efficiency (electronic supplementary material, figure S4E). We found that the filtration efficiency was optimal for tip lengths near the observed value with slight decreases for shorter and longer tip lengths (6% increase in cut-off diameter for 1.0 mm length, 8% increase for 2.4 mm, compared with measured 2.0 mm).

Given the effects of the lobe tip angle and length on the flow and performance of the filtering structure, we next considered how the sharpness of the lobe tip influenced flow separation and the flow fields around the filter. The tip radius was varied while holding other morphological parameters constant (measured: 0.22 mm, examined: 0.05–0.35 mm), and the flow over the filter lobes in the wing-like orientation was simulated (electronic supplementary material, figure S5). We found that sharp filter lobes induced the formation of a large captive vortex immediately beneath the convexity on the downstream surface of the filter lobe (electronic supplementary material, figure S5A–C). As the tip radius was increased, this captive vortex tended to decrease in size and shift upwards within the pore. This change in flow pattern produced unexpectedly small changes in the hydrodynamic permeability of the structure (16% decrease for 0.35 mm compared with 0.05 mm tip radius, electronic supplementary material, figure S5D). However, as the tip radius increases the estimated filtering efficiency was observed to decrease substantially (94% increase in cut-off particle diameter for 0.35 mm compared with 0.05 mm tip radius, electronic supplementary material, figure S5E).

In contrast to the large effects lobe tip radius had on the flow patterns around filters in the wing-like orientation, the lobe tip radius had modest effects on the flow through filters in the spoiler-like orientation (electronic supplementary material, figure S6A–C). We observed that when the filter lobes were sharp, a captive vortex was formed immediately beneath the lobe apex. As the radius of the tip was increased, this captive vortex was found to shift upwards to a position directly between the apex of the filter lobe and the convex angle on the opposing surface of the adjacent filter lobe. In all cases, the separation point was on the apex of the filter lobe at the location protruding furthest into the freestream flow. Increases in the lobe tip radius were found to produce moderate increases in the hydrodynamic permeability of the structure (51% increase for 0.35 mm compared with 0.05 mm tip radius, electronic supplementary material, figure S6D). However, increases in the lobe tip radius had smaller effects on the estimated filtration efficiency (22% increase in cut-off particle diameter for 0.35 mm compared with 0.05 mm tip radius, electronic supplementary material, figure S6E).

Together, these results support the conclusion that the shape of the lobe tip strongly influences the location of flow separation, formation of captive vortices, and filtration efficiency.

### (e) Flow stability is determined by freestream flow and filter pressure

**Figure 6.**
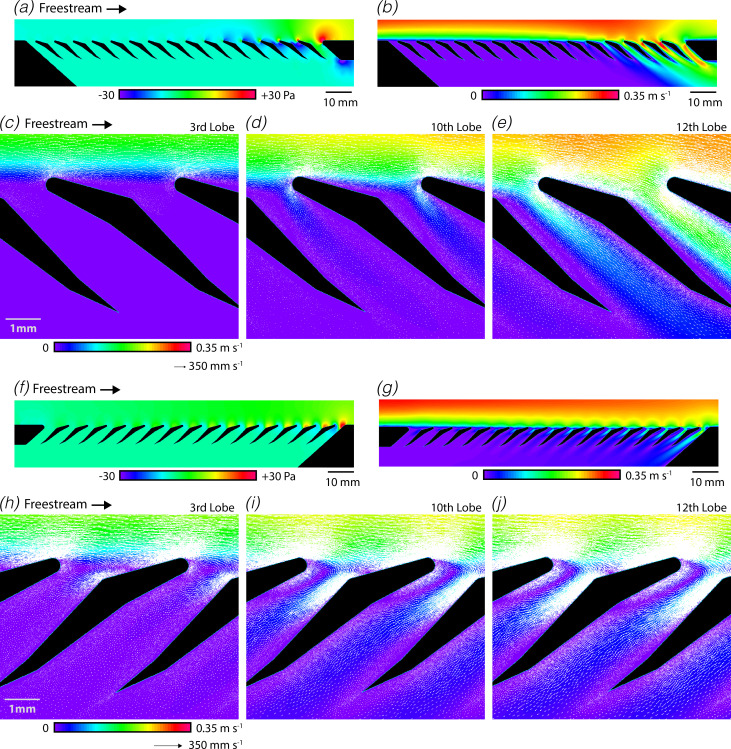
Filters in wing-like orientation exhibit substantial heterogeneity along the array. (*a*) Pressure field around the filter lobes (flow from left to right). (*b*) Velocity magnitude around filter lobes. (*c–e*) Flow fields around the filter lobes depicted by velocity magnitude (background) and flow vectors (white arrows) for third, tenth and twelfth lobes in the array. (*f–j*) Similar to (*a–e*) except show for spoiler-like orientation.

Our results indicate that the mobulid filtering structure is more sensitive to morphological variation when in the wing-like orientation compared with the spoiler-like orientation. To understand this sensitivity, we conducted additional simulations examining different regions on the filter and flow conditions. Each of the prior simulations examined the flow and filtration performance at a single pore near the middle of the array (at the tenth lobe) to aid comparisons. We next compared the flow fields around upstream versus downstream filter lobes (figure 6). We found that filters in the wing-like orientation exhibited substantial variation in both the pressure distribution and flow patterns around the filter lobes. Filter lobes on the downstream side of the array produced negative pressures within the pore (figure 6*a*) that were associated with high transverse velocities (figure 6*b*) and reduced captive vortex sizes (figure 6*c*–*e*). Although boundary layer development effects might be expected to produce some anomalies at the very beginning or end of the array, it was surprising that variation was observed near the middle of the array. In comparison, less heterogeneity was observed for filters in the spoiler-like orientation. Although the flow rate through the filter was modestly increased at downstream positions on the array (figure 6*f*–*g*), the qualitative form of the flow around the filter lobes remained similar (figure 6*h*–*j*).

To further understand this process, we next sought to determine how variation in the freestream velocity influences the flow patterns around the filter. The flow around the filter was simulated for a range of freestream velocities values while holding the pressure across the filter constant. For filter lobes in the wing-like orientation, we found that the flow was highly sensitive to the freestream velocity and filter pressure. Decreased freestream velocities prevented the formation of a captive vortex and allowed flow to pass freely through the filter pore (electronic supplementary material, figure S7A–B). The transverse velocity and hydrodynamic permeability increased sharply with reduced freestream flow speeds, increasing by 5.2× for a freestream velocity of 100 mm s^–1^ compared with the 300 mm s^–1^ freestream estimated to occur for freely swimming animals (electronic supplementary material, figure S7C). The simulated filtration efficiency also decreased sharply if the freestream velocity was reduced below the estimated value (electronic supplementary material, figure S7D). For freestream velocity values greater than the estimated value, the transverse velocity approached near zero values or even reversed direction. By contrast, for filter lobes in the spoiler-like orientation, we found that decreased freestream velocities had more modest effects on both the flow patterns and the filtration efficiency (electronic supplementary material, figure S8). For example, the transverse velocity was increased for reduced freestream flow speeds, increasing by 2.0× for a freestream velocity of 100 mm s^–1^ compared with the 300 mm s^–1^ reference value (electronic supplementary material, figure S8D).

The strong effects of freestream velocity and filter pressure on the flow through wing-like filter lobes may contribute to the observed flow heterogeneity along the array. Flow passing over the array may generate local pressure gradients that induce high transverse velocities, which in turn alter the flow conditions around subsequent filter lobes. A deeper understanding of these processes and this instability is key to understanding the biomechanics of filter feeding in these animals.

## 4. Conclusion

Devil rays use a highly specialized filtering structure to filter out solid food particles from the surrounding seawater at exceptional volumetric flow rates. Prior studies have indicated that this process uses a previously undescribed filtration mechanism referred to as ricochet separation. In this mechanism, captive vortices form within the pores of the filtering structure, bringing fluid streamlines into close proximity to solid surfaces of the filter, and solid particles following these streamlines impact the surface and are pushed away from the filter pore. This study explored how morphological changes to the filter affect filtration performance. Our results indicate that the factors which determine the performance of devil ray filters have little overlap with other types of filters. For example, the permeability and filtration efficiency of a sieve filter are determined almost exclusively by the pore width, but our results indicate that pore width is largely irrelevant to the performance of the mobulid filter. Instead, our results suggest that angle and shape of the leading edge of the filter lobes have pronounced effects on the flow patterns and filtration efficiency. This study also raises important unresolved questions. Our findings indicate that the flow and filtration efficiency are heterogenous over the filter lobe array for filters in the wing-like orientation, and that this heterogeneity strongly affects the properties of the filter. Studies aimed at understanding the complex interplay between the filter morphology, surrounding flow conditions and this heterogeneity are likely to generate important insights in the anatomy and biomechanics of filter feeding in these animals. Overall, these findings provide a critical step towards understanding how morphological differences between mobulid rays affect their filtration performance and diet preferences. In addition, this work provides a template for further optimization of bioinspired engineered filters.

## Data Availability

Scripts and model files have been deposited in a public repository [31]. Supplementary material is available online [34].
